# Prevalence of temporomandibular disorder in irritable bowel syndrome (IBS) patients: a systematic review

**DOI:** 10.22514/jofph.2025.026

**Published:** 2025-06-12

**Authors:** Michele Cricri, Gaetano Luglio, Francesca Tropeano, Antonio Miele, Giovanni Domenico De Palma, Maria Maddalena Marrapodi, Diana Russo, Marco Cicciù, Giuseppe Minervini

**Affiliations:** ^1^Endoscopic Surgery Unit, Department of Clinical Medicine and Surgery, University of Naples “Federico II”, 80131 Naples, Italy; ^2^Endoscopic Surgery Unit, Department of Public Health, University of Naples “Federico II”, 80131 Naples, Italy; ^3^Endoscopic Surgery Unit, Department of Advanced Biomedical Sciences, University of Naples “Federico II”, 80131 Naples, Italy; ^4^Department of Woman, Child and General and Specialist Surgery, University of Campania “Luigi Vanvitelli”, 80138 Naples, Italy; ^5^Multidisciplinary Department of Medical-Surgical and Dental Specialties, University of Campania “Luigi Vanvitelli”, 80138 Naples, Italy; ^6^Department of Biomedical and Surgical and Biomedical Sciences, Catania University, 95123 Catania, Italy; ^7^Saveetha Dental College and Hospitals, Saveetha Institute of Medical and Technical Sciences (SIMATS), Saveetha University, 600077 Chennai, India

**Keywords:** Temporomandibular disorders, Irritable bowel syndrome, Prevalence, Systematic review, Meta-analysis, Psychosomatic disorders

## Abstract

**Background**: Temporomandibular disorders (TMD) refer to a collection of pathological conditions that impact the stomatognathic system, often associated with psychiatric comorbidities. Interestingly, previous studies have reported a higher prevalence of TMD in individuals affected by irritable bowel syndrome (IBS), a condition commonly linked to stress-induced psychosomatic factors. The aim of this systematic review is to clarify the prevalence of TMD in IBS-diagnosed patients. **Methods**: A systematic search was conducted in PubMed, Scopus, Web of Science and Cochrane Library databases up to May 2024, following Preferred Reporting Items for Systematic Reviews and Meta-Analyses (PRISMA) guidelines and the Cochrane Handbook for Systematic Reviews of Interventions. Eligibility criteria included studies reporting prevalence data on TMD in IBS patients. Study quality was assessed using the Risk of Bias in Non-randomized Studies-of Exposure (ROBINS-E) tool. **Results**: A total of five studies, involving 3138 patients, were included. TMD prevalence was significantly higher in IBS patients compared to pooled controls. However, no significant differences were observed among IBS subtypes regarding TMD prevalence. **Conclusions**: This review highlights a substantially higher prevalence of TMD in IBS patients compared to the general population, suggesting a shared pathophysiological mechanism likely linked to stress response systems. These findings underscore the need for a multidisciplinary approach to patient management and emphasize the importance of further research to explore the causal links and underlying mechanisms between IBS and TMD. **The PROSPERO Registration**: The protocol has been registered on the International Prospective Register of Systematic Reviews (PROSPERO) with the number CRD42024542233.

## 1. Introduction

Temporomandibular disorders (TMD) represent a group of conditions causing pain and functional disability in the orofacial region, primarily through dysfunction of the masticatory muscles and temporomandibular joints [1, 2, 3, 4, 5]. These disorders affect approximately 5–12% of the population and are frequently associated with other stress-related chronic conditions [6, 7, 8, 9]. Irritable bowel syndrome (IBS), on the other hand, is a functional gastrointestinal disorder characterized by abdominal pain and altered bowel habits. It is often accompanied by chronic pain and psychosocial distress, affecting a significant portion of the global population [10, 11, 12]. Early studies suggesting a possible relationship between these two conditions report a higher prevalence of TMD in IBS patients compared to the general population [13, 14, 15, 16, 17, 18]. This correlation points to a shared pathophysiological mechanism, potentially involving dysregulation of the hypothalamic-pituitary-adrenal (HPA) axis, which governs stress responses and modulates nociception [19, 20, 21, 22, 23].

A recent systematic review of 21 cohort studies identified several factors contributing to the onset of TMD, with IBS being one of them [24]. These findings support the hypothesis that shared mechanisms, such as heightened pain sensitivity and psychological distress, may underpin both conditions. The literature suggests that IBS patients, like those suffering from other chronic pain syndromes (*e.g.*, fibromyalgia, chronic fatigue syndrome), exhibit symptoms overlapping with TMD, likely due to central sensitization. This phenomenon occurs when the central nervous system becomes hyperresponsive to normal sensory input, amplifying pain perception [25].

Psychological factors play a pivotal role in both IBS and TMD. High rates of anxiety and depression are observed in patients with both conditions, further complicating treatment outcomes [26, 27, 28]. Exploring the relationship between these disorders necessitates comprehensive research into the biochemical, neural and psychological pathways that may interconnect TMD and IBS. Moreover, clinical trials exploring combined therapeutic approaches that address both physiological and somatic symptoms are essential for improving management strategies.

Finally, the interaction between TMD and IBS underscores the complex interplay among gastrointestinal systems, pain modulation mechanisms and psychological factors. A multidisciplinary approach integrating gastroenterology, neurology, psychology, and pain management is indispensable for accurate diagnosis and effective treatment of these co-morbid conditions.

The aim of this article is to examine the prevalence of TMD among IBS patients (and *vice versa*). We incorporate recent research findings to shed light on the relationship between TMD and IBS, emphasizing the importance of multidisciplinary approaches for diagnosis and management, and propose directions for future research and clinical practice.

## 2. Materials and methods

### 2.1 Eligibility criteria

All documents were evaluated for eligibility using the Population, Exposure, Comparator and Outcomes (PECO) model.

(P) Participants consisted of human subjects.

(E) The Exposure consisted to patient with IBS.

(C) The Comparison was with control group.

(O) The Outcome consisted of Prevalence of Temporomandibular disorders in IBS patients.

Only papers providing data at the end of the intervention were included. Exclusion criteria were as follows: (1) patients suffering from any chronic inflammatory and rheumatic diseases (*e.g.*, juvenile idiopathic, psoriatic or rheumatoid arthritis); (2) patients with dental pain; (3) those with psychiatric illnesses; (4) patients with a history of facial trauma; (5) studies including individuals with partial prostheses; (6) studies with cross-over study design; (7) studies written in a language other than English; (8) full-text unavailability (*e.g.*, posters and conference abstracts); (9) research involving animals; (10) review articles (whether topical or systematic); (11) case reports or case series.

### 2.2 Search strategy

We conducted a systematic search of Web of Science, PubMed, Scopus, and the Cochrane Library for articles published from their inception through May 2024, using the search strategy outlined in Table 1. Additionally, we manually reviewed the references and prior systematic reviews on related topics.

This systematic review adhered to the Cochrane Handbook for Systematic Reviews of Interventions and the 2020 Preferred Reporting Items for Systematic Reviews and Meta-Analyses (PRISMA) guidelines (see the section **Supplementary material**). The protocol has been registered on the International Prospective Register of Systematic Reviews (PROSPERO) with the number CRD42024542233.

**Table 1.** Search strategy.

Database	Search strategy
PubMed	Search: (temporomandibular disorders) AND (ibs) (“temporomandibular joint disorders” [MeSH Terms] OR (“temporomandibular”[All Fields] AND “joint![All Fields] AND “disorders”[All Fields]) OR “temporomandibular joint disorders” [All Fields] OR (“temporomandibular”[All Fields] AND “disorders”[All Fields]) OR “temporomandibular disorders”[All Fields] AND “ibs”[All Fields]
Scopus	TITLE-ABS-KEY (temporomandibular AND disorders AND ibs)
Web of Science	(ALL=(temporomandibular disorders)) AND ALL=(ibs)
Cochrane Library	(Temporomandibular disorders) OR (TMD) AND (Irritable bowel syndrome) OR (IBS)

*IBS: irritable bowel syndrome.*

### 2.3 Data extraction

Data from the included studies were extracted by two reviewers (GL and GM) using a customized Microsoft Excel sheet. Any disagreements were resolved by consensus with a third reviewer (MC). Extracted data included: (1) First author; (2) Year of publication; (3) Nationality; (4) Number of study participants; (5) Diagnostic tool; and (6) Clinical relevance.

### 2.4 Quality assessment

Two reviewers (GL and GM) assessed the risk of bias using the Risk of Bias in Non-randomized Studies-of Exposure (ROBINS-E) tool. This tool offers a systematic method for evaluating the risk of bias in observational epidemiological studies, encompassing seven distinct domains of bias. Each domain is evaluated using a series of signaling questions designed to collect information about the study and its analysis. Once the relevant signaling questions are answered, three summary judgments are made for each domain. These judgments are then combined to produce an overall assessment of the risk of bias. Any disagreement was discussed, until a consensus was reached, with a third reviewer (MC).

### 2.5 Quality assessment and risk of bias

Version 2 of the Cochrane risk-of-bias tool for randomized trials (RoB 2) was utilized by two reviewers (GL and GM) to evaluate the risk of bias in the included studies. This widely recognized tool evaluates the quality of randomized trials by analyzing six key domains of potential bias: random sequence generation, allocation concealment, participant and personnel blinding, outcome assessment blinding, handling of incomplete outcome data and selective reporting. Disagreements were resolved through discussion, with a third reviewer (MC) mediating when necessary.

### 2.6 Statistical analysis

We conducted the pooled analysis with Review Manager, version 5.2.8 (Cochrane Collaboration, Copenhagen, Denmark; 2014). Heterogeneity among the studies was assessed using the Higgins Index (*I*^2^) and the chi-square test. The levels of heterogeneity were categorized as follows: low (<30%), medium (30–60%) and high (>60%).

## 3. Results

### 3.1 Study characteristics

A total of 102 studies met the initial search criteria from the selected sources. A PRISMA 2020 flow diagram (Fig. 1) illustrates the process, highlighting that, based on strict pre-defined study selection criteria, only five studies were ultimately included in this systematic review. Initially, 55 articles were excluded: 49 review papers, 4 animal studies and 2 non-English publications. The remaining 81 articles advanced to the second phase, which involved screening titles and abstracts to determine alignment with the PECO criteria. Of these, 32 were identified as duplicates and removed. Among the 49 remaining records, 44 were excluded for not addressing the PECO question. Consequently, 5 studies were included in the final analysis. These studies compared the prevalence of temporomandibular disorders (TMD) in individuals with irritable bowel syndrome (IBS) to those without IBS. Table 2 (Ref. [29, 30, 31, 32, 33]) below synthesizes the studies included in the review.

**Fig. 1.** PRISMA Flowchart. PRISMA: Preferred Reporting Items for Systematic Reviews and Meta-Analyses; PECO: Population, Exposure, Comparator and Outcomes.

**Table 2.** Principal elements of the included studies.

Author	Year	Nationality	Number of study participants	Diagnostic tool	Clinical relevance
Korszun *et al*. [29]	1998	USA	92 subjects	Report of past diagnosis (TMD, IBS)	Chronic facial pain often coexists with stress-related syndromes, possibly due to shared dysfunction of the hypothalamic-pituitary-adrenal stress hormone axis in predisposed individuals.
Aaron *et al*. [30]	2000	USA	94 subjects	RDC/TMD Criteria (TMD) 1978 Manning Criteria (IBS)	Patients with TMD share key symptoms with those affected by Chronic fatigue syndrome and Fibromyalgia. Several systemic conditions frequently co-occur with TMD.
Mobilio *et al*. [31]	2019	Italy	82 subjects	RDC/TMD Criteria (TMD) Rome III Criteria (IBS)	TMD patients have a greater risk of having IBS symptoms compared to healthy controls. TMD patients present also more severe forms of IBS than control population.
Sanders *et al*. [32]	2013	USA	2722 subjects	RDC/TMD Criteria (TMD) Rome III Criteria (IBS)	This article explores health conditions commonly associated with TMD, noting that first onset TMD was three times more frequent in patients with IBS than those without .
Gallotta *et al*. [33]	2017	Italy	148 subjects	RDC/TMD Criteria (TMD) Rome III Criteria (IBS)	IBS patients had over three times the risk of TMD compared to healthy controls, with those meeting TMD also showing chronic facial and abdominal pain, psychiatric comorbidities and a higher prevalence in females.

*TMD: temporomandibular disorders; IBS: irritable bowel syndrome; RDC/TMD: research diagnostic criteria for temporomandibular disorders.*

### 3.2 Main findings

Finally, five articles were included in the systematic review, and each gave quite specific information on the possible association of temporomandibular disorders (TMD) with irritable bowel syndrome (IBS):

Korszun *et al*. [29] (1998), Aaron *et al*. [30] (2000) and Mobilio *et al*. [31] (2019)—These studies outlined a high prevalence of IBS among the patients diagnosed with TMD themselves. Indeed, out of these findings, Korszun *et al*. [29] reported a 46% overlap of the two conditions; Aaron *et al*. [30] reported a 64% prevalence of IBS among patients with history of TMD, compared to 18% among healthy controls (*p* < 0.001); similarly, in the study by Mobilio *et al*. [31], 46.8% of TMD patients were diagnosed with IBS, whereas only 11.4% of healthy controls met the Rome III criteria for diagnosis of IBS. This large coexistence of these conditions may suggest a shared etiological relationship, comorbidity, or more likely, a common pathophysiology of the two conditions.

Sanders *et al*. [32] (2013)—This prospective study concluded that the incidence of first onset TMD was three times higher in subjects with IBS than in those without IBS symptoms at enrollment (Hazard Ratio (HR) 3.00, 95% Confidence Interval (CI): 1.85–4.84). It highlighted the necessity of using standardized diagnostic criteria in the field of IBS and TMD for a clearer relationship of the two diseases and their proper management.

Gallotta *et al*. [33] (2017)—According to this study, the risk of TMD in patients with IBS could be quantified at 3.41 times the risk in the healthy population (Odds Ratio (OR) = 3.41; 95% CI: 1.66–7.01). In particular, TMD was diagnosed in 54.9% of IBS patients, while only in 26.3% of healthy controls (*p* = 0.001). It was ascertained that the risk of TMD did not change among the IBS subtypes—IBS-D (diarrhea), IBS-C (constipation) and IBS-M (mixed-type). The study further indicated that TMD patients also reported chronic facial and abdominal pain and psychiatric disorders, with a female predominance.

Each of these studies makes it possible to think that IBS and TMD could have common etiological factors or modulate the pathogenesis of the other. The only consistent findings across these studies were that the prevalences of TMD were significantly higher in patients with IBS than in the general population and *vice versa*. This once more highlights the necessity of a multidisciplinary approach in the treatment for both gastrointestinal and musculoskeletal symptoms.

### 3.3 Meta-analysis

In this study we performed two statistical analyses. The first analysis concerns the prevalence of IBS in patients with TMD. In this statistical analysis we considered Aaron and Mobilio’s study; the third study (Korszun) was excluded due to lack of a control group. The second statistical analysis concerns the prevalence of TMD in patients with IBS. Two studies were considered in this statistical analysis.

The first meta-analysis, as shown in the forest plot in Fig. 2, assesses the prevalence of IBS.

**Fig. 2.** Forest Plot, evaluating the risk of IBS in patients with TMD. TMD: temporomandibular disorders; *I*^2^: Higgins Index; M-H: Mantel-Haenszel analysis; CI: Confidence Interval.

The meta-analysis was conducted by fixed effect model because of the low heterogeneity (*I*^2^ = 0%) among the two included studies that compared the prevalence of IBS in TMD patients and control subjects (individuals not exposed to TMD). The overall effect, reported in the forest plot (Fig. 2), revealed that subjects exposed to TMD had a higher prevalence of IBS signs and symptoms than controls (Relative Risk (RR): 3.82; 95% CI: 1.94–7.52; *Z* = 3.88; *p* = 0.0001), implying a significant positive association between IBS and TMD. Patients with TMD have an increased risk of developing IBS.

The second meta-analysis, as shown in the forest plot in Fig. 3, assesses the prevalence of TMD in patients with IBS.

**Fig. 3.** Forest Plot, evaluating the risk of TMD in patients with IBS. IBS: irritable bowel syndrome; *I*^2^: Higgins Index; M-H: Mantel-Haenszel analysis; CI: Confidence Interval.

The meta-analysis was performed using a random-effects model due to the high heterogeneity (*I*^2^ = 97%) observed between the two studies that compared the prevalence of TMD signs and symptoms in IBS patients versus control subjects (individuals without IBS). The overall effect, reported in the forest plot (Fig. 3), revealed that subjects exposed to TMD had a higher prevalence of IBS than controls (RR: 3.01; 95% CI: 2.03–4.47; *Z* = 5.46; *p* < 0.00001), implying a significant positive association between TMD and IBS.

### 3.4 Quality assessment and risk of bias

Figs 4,5 illustrate the risk of bias for the studies included in the review. All studies demonstrated a low risk of bias in the randomization process and allocation concealment. Neither study excluded a performance; however, a medium risk of performance bias was observed across the studies. In general, the risk of bias was low across all studies.

**Fig. 4.** Bias Plot. +: Low risk of bias; −: High risk of bias.

**Fig. 5.** Bias assessment.

## 4. Discussion

The interaction between temporomandibular disorders (TMD) and irritable bowel syndrome (IBS) reveals a complex interplay of physical and psychological factors. A growing body of evidence supports the notion that these conditions are interconnected, with each potentially influencing the onset or progression of the other. The findings from studies included in this review provide compelling evidence for a significantly higher prevalence of TMD in IBS patients and *vice versa*, emphasizing the need for a deeper understanding of their shared mechanisms.

Indeed, both TMD and IBS are considered complex disorders with overlapping features, including heightened pain sensitivity and central sensitization, which may be secondary to a dysregulation of the hypothalamic-pituitary-adrenal (HPA) axis—a critical regulator of stress responses. The HPA axis dysfunction results in altered nociceptive processing and increased susceptibility to stress, which exacerbates symptom severity and chronicity in both disorders [34].

Additionally, studies reviewed in this analysis consistently report a strong association between these psychological conditions and the co-occurrence of TMD and IBS. High rates of anxiety and depression in patients with both disorders not only complicate treatment but also highlight the influence of the gut-brain and brain-pain axes in their pathogenesis. This underscores the importance of addressing psychological factors and incorporating psychological and behavioral therapies in clinical management to achieve more effective and lasting outcomes [35].

Interestingly, a strong female predominance has been demonstrated among patients with both TMD and IBS, consistent with the broader epidemiological data on chronic pain and functional gastrointestinal disorders. This gender disparity may reflect hormonal influences on pain sensitivity, stress responses and immune regulation [36].

Traditionally, these disorders have been treated within separate medical specialties—gastroenterology for IBS and dentistry for TMD. However, a multidisciplinary approach is crucial to understanding their complex, interrelated causes and to developing comprehensive management plans addressing the full range of symptoms. This approach should include dietary modifications, medications, physical therapy, and psychological interventions targeting shared physiological pathways [37].

Recognizing the high comorbidity between TMD and IBS can enhance diagnostic accuracy, reducing the risk of misdiagnosis and delays in treatment. Routine screening for both conditions in patients presenting with primary symptoms can lead to earlier intervention and improved clinical outcomes.

Future research should focus on the intricate pathophysiological links between TMD and IBS, exploring genetic, environmental and psychosocial factors that contribute to their coexistence. Identifying new therapeutic targets will be essential in alleviating the burden of these chronic conditions and improving patient quality of life.

## 5. Conclusions

In summary, this review emphasizes the connection between irritable bowel syndrome (IBS) and temporomandibular disorder (TMD). Our comprehensive analysis of TMD prevalence in IBS patients reveals a complex, intertwined relationship that calls for a holistic approach to managing these conditions. Studies consistently show a higher prevalence of TMD among individuals with IBS compared to the general population, with rates significantly exceeding those in individuals without IBS. Our findings confirm that IBS patients face over three times the risk of developing TMD across various IBS subtypes (constipation, diarrhea and mixed), a trend also observed by other researchers. This association remains consistent across demographic factors such as age and sex.

The underlying links between these conditions stem from shared neurological, psychological and physiological dysregulations. Patients with both IBS and TMD often present with elevated depression levels and greater chronic pain severity, suggesting the involvement of central sensitization and hypothalamic-pituitary-adrenal axis dysregulation. These findings highlight the importance of a biopsychosocial approach to assessment and treatment, addressing not only physical symptoms but also psychological factors.

Additionally, a gender predisposition is evident, with a notable predominance of females affected by both conditions. This suggests potential hormonal or other sex-specific biological factors influencing susceptibility and manifestation of these disorders.

Looking ahead, further research is needed to explore the intricate relationships between gastrointestinal and musculoskeletal disorders. Future studies should investigate the underlying mechanisms in greater depth, which will be crucial for developing targeted, multidisciplinary interventions.

## 6. Limitations

The results of this review must be considered in the context of several limitations. The small sample sizes and variability in study designs of the analyzed studies, including differences in diagnostic criteria for TMD and IBS over time, may limit the generalizability of results. Additionally, the heterogeneity observed in meta-analyses underscores the need for standardized methodologies in future research. Larger, well-designed studies with robust criteria are essential to validate the reported associations and refine our understanding of the TMD-IBS connection.

## Supplementary Material

Supplementary material associated with this article can be found, in the online version, at https://files.jofph.com/files/article/1933034418900746240/attachment/Supplementary%20material.docx.


